# The Community Oncology and Academic Medical Center Alliance in the Age of Precision Medicine: Cancer Genetics and Genomics Considerations

**DOI:** 10.3390/jcm9072125

**Published:** 2020-07-06

**Authors:** Marilena Melas, Shanmuga Subbiah, Siamak Saadat, Swapnil Rajurkar, Kevin J. McDonnell

**Affiliations:** 1 The Steve and Cindy Rasmussen Institute for Genomic Medicine, Nationwide Children’s Hospital, Columbus, OH 43205, USA; Marilena.Melas@nationwidechildrens.org; 2 Department of Medical Oncology and Therapeutics Research, City of Hope Comprehensive Cancer Center, Glendora, CA 91741, USA; ssubbiah@coh.org; 3 Department of Medical Oncology and Therapeutics Research, City of Hope Comprehensive Cancer Center, Colton, CA 92324, USA; ssaadat@coh.org; 4 Department of Medical Oncology and Therapeutics Research, City of Hope Comprehensive Cancer Center, Upland, CA 91786, USA; srajurkar@coh.org; 5 Department of Medical Oncology and Therapeutics Research, City of Hope Comprehensive Cancer Center and Beckman Research Institute, Duarte, CA 91010, USA; 6 Center for Precision Medicine, City of Hope Comprehensive Cancer Center, Duarte, CA 91010, USA

**Keywords:** community oncology, academic cancer center, precision medicine, cancer genetics, cancer genomics

## Abstract

Recent public policy, governmental regulatory and economic trends have motivated the establishment and deepening of community health and academic medical center alliances. Accordingly, community oncology practices now deliver a significant portion of their oncology care in association with academic cancer centers. In the age of precision medicine, this alliance has acquired critical importance; novel advances in nucleic acid sequencing, the generation and analysis of immense data sets, the changing clinical landscape of hereditary cancer predisposition and ongoing discovery of novel, targeted therapies challenge community-based oncologists to deliver molecularly-informed health care. The active engagement of community oncology practices with academic partners helps with meeting these challenges; community/academic alliances result in improved cancer patient care and provider efficacy. Here, we review the community oncology and academic medical center alliance. We examine how practitioners may leverage academic center precision medicine-based cancer genetics and genomics programs to advance their patients’ needs. We highlight a number of project initiatives at the City of Hope Comprehensive Cancer Center that seek to optimize community oncology and academic cancer center precision medicine interactions.

## 1. Introduction

Historically, the practice and delivery of healthcare in the community contrasted significantly with medical care provided at the academic medical center [1,2]. These differences manifested across specialty practices, including oncology [3,4]. Rapid advances in molecular diagnostics, the advent of targeted therapies and the introduction of precision medicine amplified differences between community and academic oncology practices [5,6]. Reversing this historical divide, however, new financial realities, public policy initiatives and legislative mandateshave forced community oncologists and academic cancer centers to more closely align their healthcare efforts [7]. This forced alliance has lessened the separation between community and academic oncology practices and permitted broader access and utilization of precision medicine-based cancer genetics services and tumor genomic analyses. The alliance between community and academic oncology expands the capabilities and effectiveness of the community practitioner, reinforces the mission of the academic cancer center and, ultimately, secures better oncologic care for the cancer patient.

## 2. The Emergence and Evolution of the Community Health Care and Academic Medical Center Alliance

A number of key distinctions differentiate the medical care provided at community health centers (CHCs) versus academic health centers (AHCs); these differences result in complementary advantages. The overwhelming majority of patients receive their healthcare through CHCs; the CHC patient population typically exhibits great diversity across economic, racial, ethnic and social spectra [8]. CHCs offer their patients increased accessibility and enhanced client engagement [9]. In contrast, AHCs, characteristically, have focused on specialty medical care, biomedical research, the education and training of health care professionals and the stopgap provision of health care to uninsured and destitute populations [10]. These activities underlie the strengths of AHCs. These strengths include the presence of medical expertise, scientific innovation and clinical trial availability; additionally, AHCs possess unique physical resources such as libraries, computerized database management and informatics infrastructure, research laboratories and emergency room facilities [11,12]. Leveraging these strengths, AHCs have established their reputations and acquired leadership roles in shaping medical care and policy [10].

Until two decades ago, CHCs and AHCs functioned largely in parallel, without administrative or operational intersection. A variety of recent economic, social and regulatory circumstances, however, diminished the independence of AHCs. With the rise of community-based health care markets, particularly managed care plans, many of the operations traditionally carried out at AHCs shifted to CHCs; this shift often undercut the previously reliable revenue streams of AHCs. This situation forced reconsideration of the AHC financial model and provided impetus for the implementation of more efficient, cost-effective health care delivery strategies [13,14,15,16,17,18]. At the same time, governmental funding agencies, to ensure faithful representation of population diseases, placed a premium on the inclusion of community patients into research protocols. These agencies also issued directives to AHCs to provide comprehensive population care and mandated the formal reporting of AHC involvement with community patient populations [19,20,21,22,23,24,25]. Overall, these influences forced AHCs to redefine their core mission with a new emphasis on the integration of the CHC and their patient populations [26,27,28]. Given their previous work in shaping medical policy, their stewardship of medical education, and their diverse and extensive resources, AHCs readily assumed a leadership role in the restructuring of the CHC/AHC relationship and the creation of integrated partnerships [2,29,30,31,32,33,34,35].

The alliance between CHCs and AHCs provides advantages to both partners. CHCs and AHCs enjoy better positioning within the healthcare marketplace. The improved marketplace positioning results primarily from economy of scale pricing that accompanies the integration and expansion of patient services, procedures and therapeutics; the alliance secures for both partners more stable financial footings [36]. The alliance makes possible specific benefits for the CHC. This alliance permits the CHC more direct access to AHC-generated experimental therapeutics, clinical trials, translational research, medical devices and protocols [37]. Further, evidence suggests that affiliation with an AHC often enhances the prestige and attractiveness of the CHC, increases patient and clinical staff retention, fosters more opportunity for continuing professional development, frequently results in greater professional satisfaction and has the potential to enhance the quality and efficacy of the CHC [38,39,40]. For the AHC, partnerships with a CHC allow for enhanced opportunities to interact more tangibly with the community patient population and expand and diversify patient pools for translational research and clinical trial enrollment; partnerships also increase the ability of AHCs to mitigate outcomes and patient access disparities [41]. Multiple examples of successful CHC/AHC partnerships exist; they serve as models for the feasibility and potential future CHC/AHC partnerships [42,43,44].

## 3. Community Oncology and Academic Cancer Center Alliance

The integration of CHCs with AHCs most tangibly manifests as practice changes within specific departments, including, prominently, medical oncology [45,46,47,48,49,50,51]. During recent years cancer care has transitioned from primarily private, CHC-based oncology practices to AHC-affiliated and -integrated network cancer centers [51,52,53,54,55]. This transition has advantaged the community cancer patient as the services associated with the academic cancer center provide added value. 

At the City of Hope Comprehensive Cancer Center (COHCCC), patients identify a number of key value elements associated with the academic cancer center including access to cancer disease specialists, the availability of clinical, translational and basic science researchers, potential for clinical trial participation and enhanced comprehensive care coordinated through multidisciplinary clinical teams [56]. 

Across a broad range of cancers, patients experience improved survival when receiving treatment at an academic cancer center or at a community hospital associated with an AHC [57,58,59,60,61,62,63,64]. Academic cancer centers provide additional value to community practices through the discovery and provision of novel drugs, experimental medical devices, treatment protocols and technological advancements [65,66,67,68,69,70,71]. Reciprocally, academic cancer centers benefit from their alliance with community oncology practices by expanding clinical trial portfolios [72,73,74], increasing patient diversity in cancer translational and basic research initiatives [75,76,77,78,79], enhancing cancer center core mission accomplishment through community cancer patient engagement [80] and reducing cancer care costs resulting from increased patient volumes [81]. 

The introduction of new technologies and scientific techniques underscores the importance and potential of the alliance between community oncology practices and academic cancer centers. Specifically, recent advances in genetics and tumor genomics have provided a foundation for the emergence of precision oncology; the community/academic oncology alliance promises to accelerate significantly the clinical utility of precision oncology for the cancer care of community patients [82,83,84,85].

## 4. The Age of Precision Oncology

Cancers exhibit highly complex genomic and epigenomic alterations; these alterations dictate their overall phenotypic behavior that includes growth characteristics, metastatic potential, interplay between cells and microenvironmental interactions and responses. Over the past several decades, scientific strategies to prevent, diagnose and treat cancer have radically shifted from histology-based to genomically- and immunologically-informed approaches [86].

Since completion of the Human Genome Project in 2003 [87], a series of convergent technological advances resulted from academic-based initiatives. These advances include the introduction and adoption of next generation nucleic acid sequencing (NGS), exponential improvements in computer hardware capabilities, optimization of data processing approaches, evolution of increasingly sophisticated computational biological methods and the discovery and utilization of targeted cancer therapies. Together these advances made possible precision medicine and, more exactly, precision oncology [82,88,89,90].

NGS arose from innovative DNA sequencing methodologies, most notably massively parallel signature sequencing [91,92]. NGS permits tractable high throughput sequencing of immensely large and complex DNA samples such as whole human exomes and genomes [93,94]. Geneticists first employed NGS to sequence accurately and rapidly the human germline genome [95], allowing insights into the cause of inherited disease [96,97]; investigators then extended the technology to sequence somatic cancer genomes [98]. Scientists further refined the applications of NGS technology. New applications permitted assessment of not only single nucleotide variation and nucleotide insertions and deletions, but also the transcriptome to assess gene expression [99,100,101], copy number variation [102], complex genomic structural variation [103], protein-DNA interactions [104], targetable epigenetic alterations [105] and epigenetic mechanisms regulating 3D genome structure [106].

In addition to examining tumor genomics, there arose an interest in understanding the immune profiles of the tumor and its microenvironment using NGS; in part, this interest developed from the recognition that tumor genomic changes frequently result in the production of unique, highly immunogenic neoantigens that render the tumor vulnerable to immune surveillance and destruction [107,108]. With the appreciation that the immune system plays an important role in cancer initiation and progression, there has also occurred new interest in targeted therapies aimed at activation of the immune axis [109,110].

NGS generates enormous caches of data; use of these immense data sets for precision oncology requires ever increasing levels of computer hardware performance. Employment of Dennard scaling [111] and multicore architectures [112] have sustained exponential increases in computer chip performance [113,114,115]. Data processing innovations have included parallel algorithm implementation [116] and parallel data computing [117]; such innovations have force multiplied the efficiency and speed of computation. These approaches allow data analysts to keep pace with the ever increasing information workloads of precision oncology [118].

The realization of precision oncology required adoption of computational biological approaches. The creation of computational biology as an independent academic discipline resulted from the complexity and size of biological data sets. In the case of NGS, the sheer number of nucleotides reads, the task of aligning these reads to reference sequences, predicting functional consequences of genomic variation and the translation of these findings into clinically actionable information necessitated computational biological expertise [119,120,121,122]. Computational biological analysis now constitutes an integral element of the data workflow in precision oncology [123,124,125,126]; effective clinical translation depends inextricably upon the availability of these computational resources [127,128,129,130].

In the early 1970′s, Drs. Janet Rowley, Peter Nowell and Alfred Knudson, studying leukemia cell chromosomes under the microscope, suggested that a specific chromosomal translocation that resulted in the formation of the BCR-ABL fusion oncogene caused chronic myelogenous leukemia (CML); this observation established a foundation for clinical cancer genomics [131]. Oncogenic proteins consequently became a focus of therapeutic drug design; targeted therapies aimed to suppress the aberrant functions of these proteins in order to inhibit tumor progression [132,133].

The successful harnessing of precision therapeutics in oncology ultimately relies upon the availability and efficacy of targeted agents. The discovery that imatinib effectively treats CML harboring the BCR-ABL fusion protein [134] led to the drug’s FDA approval in 2001 [135], demonstrated the utility of targeted cancer therapy [136,137], kindled enthusiasm for the identification of other genetically vulnerable cancers and their treatments [90,138] and underscored the clinical value and potential of precision oncology [98,139,140]. Since the success of imatinib, the FDA has approved a multitude of additional therapies to target molecularly-altered cancers [141,142].

The clinical provision of precision oncology requires multidisciplinary support [143]; the complexity of this support will become more intense as precision oncology continues to undergo accelerating change [144,145,146]. AHCs possess the resources and organization to create this support structure; their alliance with CHC oncology practitioners will make precision oncology available to the larger CHC cancer population.

## 5. The Community Oncology/Academic Cancer Center Alliance in Germline Cancer Genetics

NGS and precision oncology have had a profound effect upon the practice of cancer genetics, including the evaluation and care of community patients with hereditary predisposition to cancer [147,148,149,150,151]. Until recently, genetic testing involved clinical assessment followed by sequential, single gene Sanger sequencing of suspect genes [152,153,154,155,156]. The advent of NGS brought high throughput germline multigene panel [157,158,159,160,161], whole exome [162,163,164,165,166,167] and whole genome assessment [168,169,170,171,172] to clinical cancer genetics. These platforms provide tremendous benefit to cancer genetics patients both in community oncology practices and at academic cancer centers; these advantages include increased diagnostic yield, increased speed of testing, optimized testing workflows, decreased expense and the discovery of new cancer-causing genes [173,174,175,176,177]. However, together with advantages, challenges and limitations arise; AHCs have the specialized resources to address these issues.

In accordance with the American College of Medical Genetics and the Association for Molecular Pathology guidelines, variants from clinical genetic testing fall along a spectrum ranging from pathogenic/likely pathogenic to benign/likely benign [178]; variants of uncertain significance (VUS) occur when there exists insufficient information for variant assignment to either the pathogenic or benign categories [179,180]. For pathogenic/likely pathogenic and benign/likely benign variants, genetic providers typically have the ability to communicate clear interpretation of results and to provide consensus health recommendations. As their pathogenicity remains uncertain, VUS challenge health care specialists to formulate and relay unambiguous health care instructions [181,182,183,184,185]; furthermore, VUS frequently cause confusion and anxiety for the patient [186,187,188,189,190]. VUS impose a significant clinical burden. More than one third of NGS-based cancer gene panel tests result in identification of a VUS [191]; whole exome and genome testing generate even greater numbers of VUS [192,193,194,195]. Moreover, if a patient belongs to a minority group, for whom genome annotations remain less well confirmed, VUS additionally increase [196].

Geneticists classify genes according to their penetrance, that is, how likely will a pathogenic variant of a gene cause disease [197]. For pathogenic variants of high penetrance genes, clinicians more often have firmly established guidelines that inform recommendations for patient screening and surveillance. However, for pathological variants of low penetrance genes, less definitive clinical guidelines exist. NGS-based testing results in increasing detection of pathogenic variants of low penetrance genes; this increased detection adds complexity and uncertainty to patient management [198,199].

Clinicians face another challenge when selecting NGS gene panels for genetic evaluation: they must select the composition of the gene panel that they will employ. This selection requires specialized education and training [200,201]. The cancer genetics expertise required to address this challenge remains scarce [202,203,204,205]; the wider use of NGS platforms in clinical oncology and continued technological advances has made this expertise even more scarce [206,207,208].

**Figure 1:**
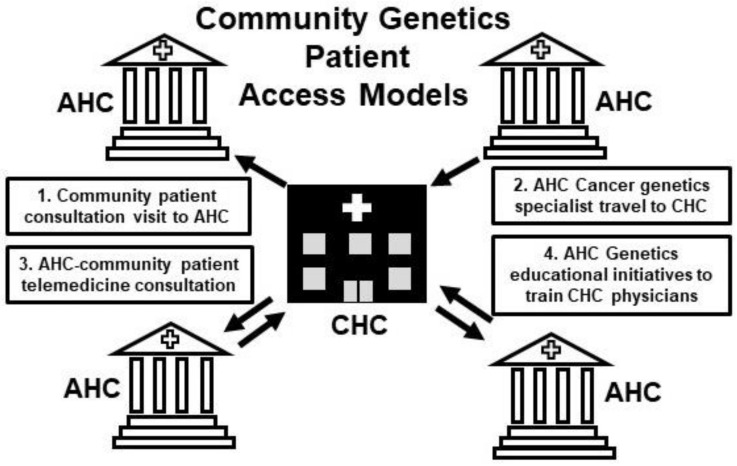
Community health center (CHC) patients requiring genetics care interface with specialists at academic health centers (AHC) through four modes of interaction. (1) The CHC patient may travel to the AHC for assessment. (2) The AHC genetics specialist may travel to a satellite CHC genetics clinic to evaluate the CHC patient. (3) CHC patients and AHC genetic specialists may interact via telemedicine consultation. (4) In order to provide genetics care to their patients, CHC physicians may undergo genetics specialty training sponsored by AHCs.

AHCs possess the clinical expertise, facilities, support personnel, and administrative structures to meet the burgeoning demands of cancer genetics and to overcome the obstacles associated with the use of NGS in the clinic. Allied community oncology practices and their patients have access to these resources and services through their partnerships with AHCs. Four access models enable community oncology patient engagement with the AHC: (1) patient consultation visits to the academic cancer center, (2) cancer genetics specialist visits to community oncology sites, (3) telemedicine- and web-based remote visits and (4) AHC-sponsored genetic education initiatives that train community oncology practitioners to assess and manage cancer genetic risk and disease (Figure 1).

Conventionally, community oncology patients have received their cancer genetics care by consulting, in person, with a specialist at an AHC [155,209,210,211]. This model disadvantages community patients who live substantial distances from an AHC as it involves significant travel time and cost commitments [210,212,213]. Alternative cancer genetics delivery models have the potential to mitigate these problems.

In the community satellite clinic model, AHC cancer genetic specialists travel to the CHC clinic on an interval basis to meet the cancer genetic needs of community patients. This approach has proven successful in a variety of circumstances where logistical or economic challenges create barriers to effective cancer genetics care [214,215,216,217].

In our digital era, innovative cancer genetics delivery models have emerged; telemedicine platforms that involve both telephony and video communication platforms represent one such model [218,219,220,221,222]. The Division of Clinical Cancer Genetics (CCG) at COHCCC has assumed a national leadership position in the adoption of digital age technologies to provide academic center cancer genetic services to community oncology practices and their patients.

The CCG formed the Cancer Screening and Program Network (CSPPN), building a bridge to community oncology practices; the CSPPN utilizes innovative videoconferencing, telemedicine and wed-based applications to provide cancer genetics services [223]. Innovation continues at the CCG with the ongoing construction of new software and web-based platforms to permit effective communication between academic cancer genetics providers and community-based patients and practitioners [224].Alongside the use of these digital platforms, the CCG has administered a landmark educational program to provide community oncology healthcare providers with the necessary training that allows them to function as competent cancer risk assessment specialists in their own communities [225]. This program, funded by the National Cancer Institute, has expanded the workforce of qualified germline genetics providers and has helped to alleviate the shortage of cancer genetics expertise in CHC practices.

Educational programs, such as that sponsored by the CCG, have acquired additional practical importance as many healthcare systems now require, prior to genetic testing, assessment by a healthcare provider trained in genetics. These requirements may hinder effective cancer genetics care, particularly in underserved communities [226]; the availability of training will help eliminate this hindrance.

## 6. The Community Oncology/Academic Cancer Center Alliance in Somatic Tumor Genomics

The use of clinical NGS in oncology has risen exponentially [227]. Hundreds of commercial and academic laboratories now offer NGS-based clinical sequencing of cancer specimens [119,228]. The NGS sequencing formats for somatic tumor sequencing include, among others, whole exome, whole genome, targeted panel, transcriptome and liquid biopsy assessments [229,230,231,232,233,234,235]. Various factors have driven the increased clinical application of NGS for somatic tumor assessment. The number of targetable genomic alterations increases substantially each year. Currently, there exist well over one hundred FDA-approved targeted therapies available for the treatment of both solid and hematological cancers [98]; over the past year alone, the FDA granted approval to nearly 20 new drugs or new indications for previously approved drugs [96]. With inclusion of therapy based upon molecular pathway considerations or off-label usage based on tissue-agnostic variant matching, the set of molecular targets and usage indications expands geometrically [236,237,238,239,240,241,242,243,244]. Purposing NGS-based somatic testing to determine clinical trial eligibility further increases the utility of NGS [245,246,247,248]; moreover, the demonstrated efficacy of testing to achieve improved outcomes has also motivated demand [248,249,250,251,252]. The decision by the Centers for Medicare and Medicaid Services to provide insurance coverage for NGS-based sequencing tests removed a financial barrier against the use of NGS, and contributed to the expanded use of this technology [253,254,255]. All told, currently over three quarters of oncologists use now NGS-base clinical testing to guide treatment decisions [256].

Significant challenges, however, temper enthusiasm for the clinical institution of somatic tumor NGS. A majority of oncologists report difficulty interpreting NGS somatic tumor testing, lack understanding of the clinical indications for testing and have inadequate opportunities to acquire the necessary training to properly use testing. One quarter of oncologists refer patients to other specialists to assist with NGS testing, and approximately 1 in 5 oncologists did not feel they had the proper knowledge to use properly NGS testing [256,257,258]. Additionally, oncologists report challenges with managing the large data volumes generated from NGS somatic testing. Oncologists also feel that they do not have the ability to distill from these reports actionable information; further, they lack the skill to manage germline variants detected as incidental findings in somatic NGS tumor testing [259,260,261,262]. These obstacles may be amplified for the CHC-based oncologist who lacks access to the necessary computational resources, logistical support and expertise in targeted therapeutics [263,264,265,266].

The CHC/AHC alliance provides solutions to alleviate these obstacles. Innovative AHC-based web applications make available to community oncologists an analytic framework and the computational tools to aid in the interpretation and clinical implementation of NGS sequencing results (Table 1). CIViC, an open access web resource, serves as a public central repository of NGS data “supporting clinical interpretations related to cancer” [267]. OncoKB, a precision oncology database, aids therapeutic decision-making based upon cancer gene variant status [268]; similarly, the web applications Personalized Cancer Therapy and My Cancer Genome assist both community and academic oncologists in selecting therapeutic options resulting from the somatic NGS of tumor specimens [269,270]. The SMART Cancer Navigator aggregates variant and clinical data from multiple data bases to assist community-based oncologists with the processing of NGS reports and the identification of effective targeted therapies [271]. At the COHCCC, investigators have configured an interactive web interface, HOPE-Genomics, that community patients and oncologists may use to better understand genomic sequencing results and treatment recommendations [224]. The COHCCC also provides to its community practice partners in-house NGS panel testing as part of its HOPESEQ molecular testing panel [272]; HOPESEQ includes genomic test reports designed to assist clinicians with interpreting the genetic testing results and clinical decision making. Furthermore, COHCCC physicians and community partners have access to Via Oncology; this tool provides a web-based clinical pathway system to help match patients with clinical trials and insurance reimbursement for NGS driven treatments [273].

**Figure 2:**
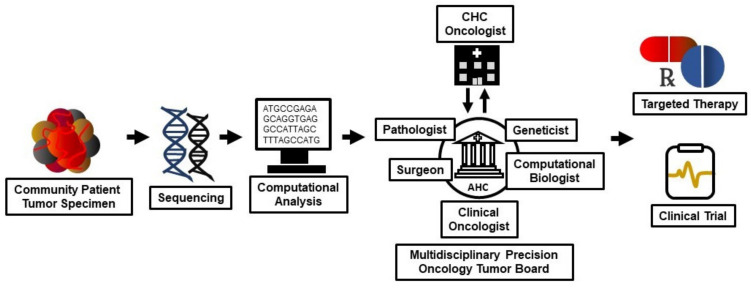
Multidisciplinary precision oncology tumor boards (POTBs) provide expert targeted drug matching and molecularly informed clinical trial enrollment for community oncology patients. Tumor specimens from community patients undergo nucleic acid sequencing with computational analysis to identify molecular alterations; this information provides a basis to discover candidate targeted therapies and determine clinical trial eligibility. An academic health center (AHC) POTB comprising, among others, clinical oncologists, pathologists, surgeons, geneticists and computational biologists, in consultation with community health center (CHC) oncologists, reviews patients’ clinical cases and their sequencing results to select appropriate targeted therapies and clinical trials.

Precision oncology tumor boards (POTBs) represent another solution to the problem of implementing NGS data in the CHC oncology clinic. POTBs arose from the need to assess, process and generate clinical treatment plans from the highly dense and complex data sets that arise from somatic NGS of tumor specimens [274]. POTBs serve two primary functions: targeted therapy drug matching and molecularly-informed clinical trial enrollment [275,276,277,278,279,280] (Figure 2).

POTBs originated within AHCs as these centers possess the multidisciplinary expertise including, among others, clinical oncologists, pathologists, genomics specialists, computational biologists, pharmacologists and clinical geneticists to efficiently identify targeted therapies and clinical trials [281,282,283,284,285,286,287,288,289,290]. Targeted therapy drug matching requires comprehensive molecular mutational profiling and downstream pathway analyses of the tumor, combined with the identification of safe and effective therapeutic agents that redress these molecular alterations [291,292,293]; CHCs typically do not possess the analytic or pharmacologic capabilities to adequately perform these activities. Most clinical trials fail [294,295,296]; these failures result from a number of factors including deficient clinical trial design, poor proof of concept planning and insufficient administrative support and compliance [297,298,299,300]. Such failures have adverse consequences for both the clinical trial sponsors as well as the patients; failure has significant economic cost and results in lost therapeutic opportunity, in addition to potentially exposing the patient to harm from the investigational protocol and drugs [301,302,303]. These clinical trial-related matters may be more acute at CHCs given their more limited resources and the absence of experienced clinical trialists [304,305,306,307]. The POTB provides appropriate, molecularly-informed clinical trial assignment for patients, maximizing both the utility of clinical trial participation and potential patient benefit [308,309,310,311,312,313,314,315,316,317,318,319,320].

Given the resource limitations of the CHC oncology clinic, community POTB operation requires innovation and dedicated planning [83,321,322]. One innovation available to community oncologists, the web-based ASCO Multidisciplinary Molecular Tumor Boards, assists oncologists with understanding precision medicine-based tumor testing and the therapy recommendations resulting from these tests [323] (Table 1). Helio Learn Genomics, another web platform, offers a number of educational modules, including POTB cases, to help providers understand the molecular bases of carcinogenesis and precision therapeutics [324]. The Pancreatic Cancer Action Network administers a Know Your Tumor program, a turn-key precision medicine initiative, that allows community oncology practitioners to submit their patients’ pancreatic cancer specimens for NGS molecular testing and to receive back a precision medicine-based treatment plan [325].

Another version of the POTB, the virtual POTB, permits the distance participation of community oncologists in an academic POTB. In this model an AHC hosts the POTB and reviews the clinical history and precision oncology testing results of the community oncology patient; subsequently, the POTB discusses with the community oncologist, using a live interactive video teleconferencing link, targeted treatment and clinical trial recommendations [263,311,326,327,328,329,330]. The Translational Genomics Research Institute (TGEN), an academic affiliate of the COHCCC, has successfully built a comprehensive, integrated, high-throughput sequencing and reporting framework that, when combined with remote teleconferencing, has proven tremendously successful in establishing efficient collaborative POTBs [331,332,333,334,335]. Together, these various models of providing clinical somatic NGS demonstrate the feasibility of leveraging precision oncology for the community-based cancer oncologist and their patients.

## 7. Conclusions

We have entered the age of precision oncology. Precision oncology offers the potential of molecularly informed medicine for the assessment of inherited cancer predisposition, as well as for the diagnosis and treatment of cancers. Realization of this potential depends upon access to specialized expertise and significant analytic and technological resources. While frequently available at AHCs, these resources have previously been limited for CHC oncology practices and their patients. In this paper, we have examined the CHC/AHC alliance and discussed examples illustrating how this alliance provides a structure that allows community cancer patients to benefit from germline and somatic precision oncology advances. Looking forward, multidisciplinary efforts, improved technology and continuing innovation promise to strengthen and facilitate the CHC/AHC alliance in oncology; this alliance offers community oncologists and their patients the prospect of unambiguous interpretation of genetic and genomic test results and optimized precision oncology care. 

## Figures and Tables

**Table 1:** Web-based genomics resources available to community oncologists.

WEB-BASED RESOURCE	URL
CIViC	civicdb.org
OncoKB	oncokb.org
Personalized Cancer Therapy	pct.mdanderson.org
My Cancer Genome	mycancergenome.org
SMART Cancer Navigator	smart-cancer-navigator.github.io/home
ASCO Multidisciplinary Molecular Tumor Boards	elearning.asco.org/product-details/multidisciplinary-molecular-tumor/boards-mmtbs
Helio Learn Genomics	healio.com
Know Your Tumor	pancan.org
